# Effect of Thermal Annealing on the Photoluminescence of Dense Si Nanodots Embedded in Amorphous Silicon Nitride Films

**DOI:** 10.3390/mi12040354

**Published:** 2021-03-25

**Authors:** Qianqian Liu, Xiaoxuan Chen, Hongliang Li, Yanqing Guo, Jie Song, Wenxing Zhang, Chao Song, Rui Huang, Zewen Lin

**Affiliations:** School of Materials Science and Engineering, Hanshan Normal University, Chaozhou 521041, China; 2018267230@stu.hstc.edu.cn (Q.L.); 2018267226@stu.hstc.edu.cn (X.C.); yqguo126@126.com (Y.G.); songjie@hstc.edu.cn (J.S.); wenxingzhang@hstc.edu.cn (W.Z.); songchao511@126.com (C.S.); rhuang@hstc.edu.cn (R.H.)

**Keywords:** photoluminescence, thin films, optical properties, SiNx

## Abstract

Luminescent amorphous silicon nitride-containing dense Si nanodots were prepared by using very-high-frequency plasma-enhanced chemical vapor deposition at 250 °C. The influence of thermal annealing on photoluminescence (PL) was studied. Compared with the pristine film, thermal annealing at 1000 °C gave rise to a significant enhancement by more than twofold in terms of PL intensity. The PL featured a nanosecond recombination dynamic. The PL peak position was independent of the excitation wavelength and measured temperatures. By combining the Raman spectra and infrared absorption spectra analyses, the enhanced PL was suggested to be from the increased density of radiative centers related to the Si dangling bonds (K0) and N_4_^+^ or N_2_^0^ as a result of bonding configuration reconstruction.

## 1. Introduction

Si nanostructures embedded in wide-bandgap dielectrics show great promise in realizing efficient Si-based light sources compatible with the mainstream complementary metal-oxide semiconductor (CMOS) technology [1,2,3,4,5,6]. Compared with the wide bandgap of silicon oxide, amorphous silicon nitride (a-SiNx) shows a narrower and tunable bandgap ranging from ~2 eV to ~5 eV. This characteristic allows more carriers to easily be injected into the Si nanostructures, thereby making a-SiNx favorable for designing stable and efficient SiN-based light-emitting devices (LEDs) at low driving voltages [7,8,9]. The structure and optoelectronic properties of Si nanostructures embedded in SiNx matrix have been extensively studied. Using high-density nanostructures is an effective way to obtain highly efficient light emission [10,11]. However, developing more efficient SiN-based LEDs is still a huge challenge. Many nonradiative recombination centers caused by structural distortions and defects in the a-SiNx matrix lead to the deterioration of luminescence process [12]. In particular, the large surface-to-volume ratio of Si nanostructures makes surface states play a more important role in the luminescence performance [13,14]. Therefore, the engineering of Si nanostructures through surface modification has become a key issue for obtaining efficient SiN-based devices with suppressed nonradiative recombination centers.

In earlier studies, we fabricated amorphous Si nanostructures with a density of over 4.6 × 10^12^/cm^2^ embedded in SiNx matrix by very-high-frequency plasma-enhanced chemical vapor deposition technique (VHF-PECVD) [11]. The dense Si nanostructures can be both optically and electrically excited and emit strong light emission. In the current work, the effect of thermal annealing on the photoluminescence of Si nanostructures embedded in SiNx was investigated. Thermal annealing notably improved the photoluminescence (PL). The enhanced PL is discussed in detail based on its annealing behavior.

## 2. Experiment

The a-SiNx films containing dense amorphous Si nanodots were fabricated by VHF-PECVD at 250 °C using the gas mixture of SiH4, NH3, and H2 as the precursor, as demonstrated previously [11]. After deposition, the films were first dehydrogenated at 400 °C and then thermally annealed at different temperatures in N_2_ atmosphere for 60 min. The microstructure of samples was characterized by the Horiba LabRAM HR Evolution Raman Spectrometer. The PL spectra of the films were measured by a Jobin Yvon FluoroLog-3 spectrophotometer equipped with a 450 W steady Xe lamp. PL decay curves were recorded on an Edinburgh FLS1000 spectrometer using a 372 nm picosecond laser (pulse width 44 ps, repetition rate = 20 MHz). The decay rates were measured at the wavelength of 570 nm. The time response of the detector and the electronics used in the PL decay measurements was less than 400 ps. The bonding configurations of the films were recorded using a Fourier-transform infrared (FTIR) spectroscope (Shimadzu IR Pretige-21).

## 3. Results and Discussion

**Figure 1:**
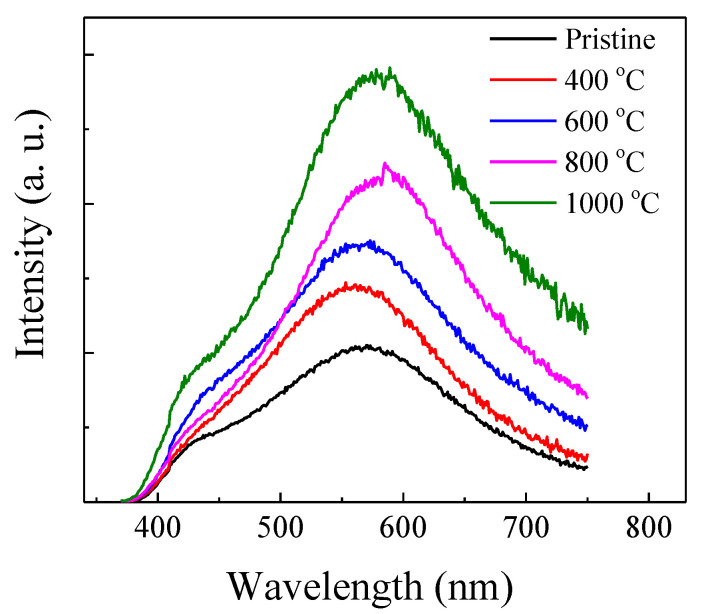
Photoluminescence (PL) spectra of the pristine and annealed samples excited by 325 nm line from a Xe lamp.

Figure 1 presents the PL spectra of the pristine and annealed samples excited by 325 nm line from a Xe lamp. The spectrum of the pristine sample showed a broad band centered at 570 nm with a shoulder at 425 nm. The spectral shape of the PL peak did not change with increasing annealing temperature. However, the PL intensity rapidly increased with increasing annealing temperature from 400 °C to 1000 °C. Compared with the pristine film, it was interesting to find that thermal annealing at 1000 °C gave rise to a significant enhancement by more than twofold in the green PL.

**Figure 2:**
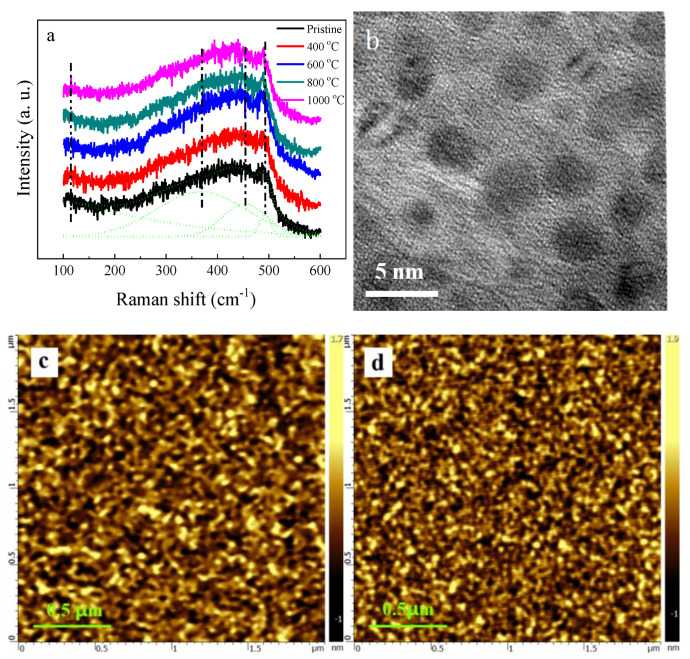
(**a**) Raman spectra of the samples with and without annealing; (**b**) Bright-field TEM image of the dense Si nanodots; (**c**) Atomic force microscopy (AFM) images of the pristine sample; (**d**) AFM images of the sample annealed at 1000 ℃.

To analyze the enhanced PL behavior, the microstructure of the samples was characterized. Figure 2a shows the Raman spectra of the samples with and without annealing. All Raman spectra displayed a broad band between 100 cm^−1^ and 600 cm^−1^. The broad Raman band can be decomposed into four peaks located at ~110 cm^−1^, ~370 cm^−1^, ~450 cm^−1^, and ~480 cm^−1^, which were attributed to the transverse-acoustic (TA), the longitude-acoustic (LA), the longitudinal-optical (LO), and the transverse-optical (TO) vibration mode of amorphous silicon, respectively [15]. This indicated that no Si nanocrystals were produced in the samples, even after they were annealed at 1000 °C. The Transmission Electron Microscope (TEM) image shown in Figure 2b further reveals that the structure of Si nanodots is amorphous. Therefore, the improved PL from the annealed samples did not result from the Si nanocrystals. As was revealed by atomic force microscopy (AFM), one can see that there was no obvious change in the surface morphology between pristine and annealed sample at 1000 ℃ (Figure 2c,d). This excluded the possibility that the enhanced PL is the result of the increase in light extraction from the annealed samples.

**Figure 3:**
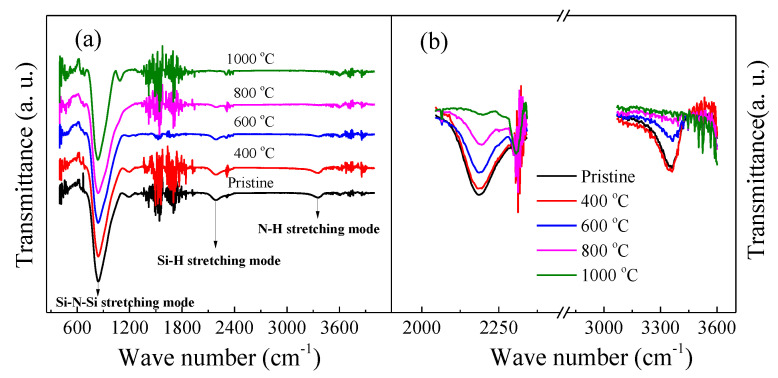
(**a**) FTIR absorption spectra of the samples; (**b**) The absorption band at ~2140 cm^−1^ and 3350 cm^−1^, respectively.

**Figure 4:**
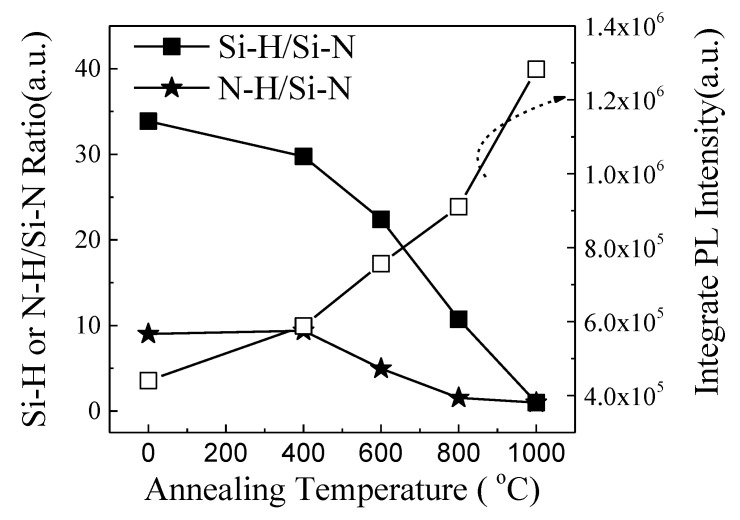
The ratios of Si–H/Si–N and N–H/Si–N deduced from the IR absorption coefficient as a function of annealing temperature.

To examine the local bonding configurations of the samples, FTIR was employed. Figure 3a displays typical FTIR absorption spectra of the samples. These spectra demonstrated the following vibrational bands. The intense absorption band at 845 cm^−1^ was associated with the Si–N stretching mode [16]. The absorption band at ~2140 cm^−1^ bands was connected to Si–H stretching mode. [16] The 3350 cm^−1^ band corresponded to the N–H stretching mode [16]. The evident feature in the FTIR spectra was that the signals of the Si–H and the N–H vibrations strongly depend on the annealing temperatures. The ratios of Si–H/Si–N and N–H/Si–N deduced from the IR absorption coefficient decreased gradually with increasing annealing temperature from 400 °C to 1000 °C, as displayed in Figure 4. Comparing the FTIR spectra with the PL spectra, it was evident that the behavior of the PL intensity with the annealing temperature exhibited a trend opposite to that of the Si–H/Si–N and N–H/Si–N ratio with the annealing temperature. This finding implied that the increase of the emission intensity was closely associated with the chemical bond reconstruction and was especially closely related to the decreasing concentration of Si–H and N–H bonds. In fact, the decrease in the concentration of Si–H and N–H bonds indicated the fracture of Si–H and N–H bonds and an increase in the concentration of Si and N dangling bonds. Therefore, the increasing concentration of Si and N dangling bonds in our case seemed to play an important role in the significant enhancement in the 570 and 425 nm PL.

**Figure 5:**
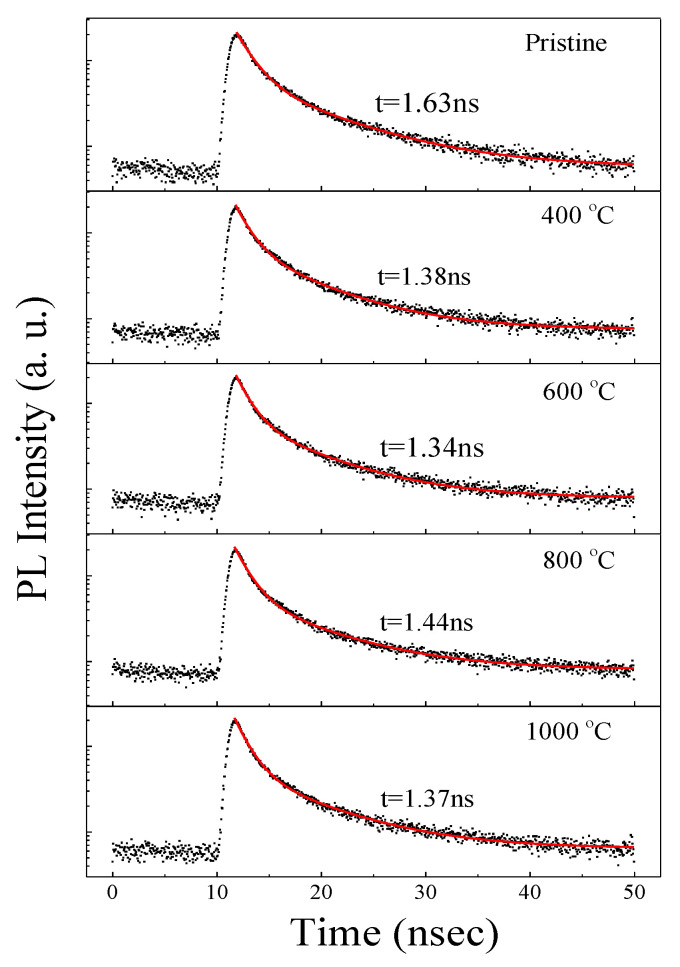
PL decay curves for all samples.

**Figure 6:**
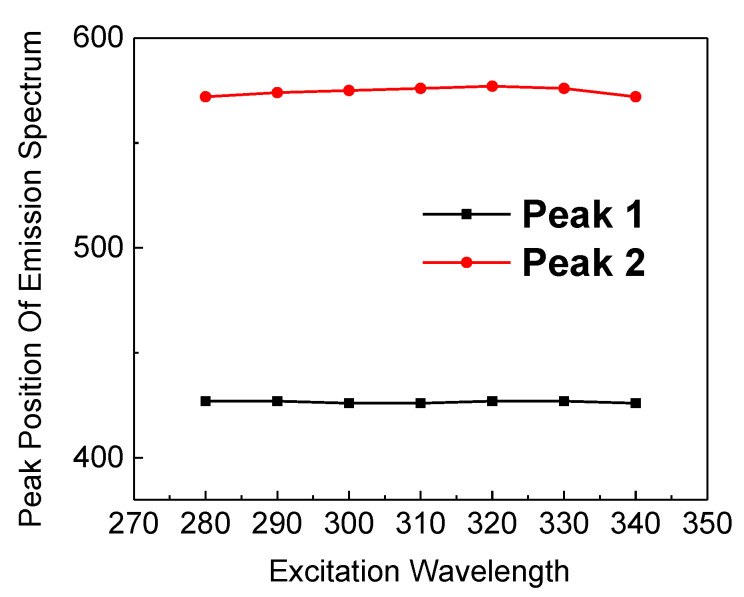
The PL positions as a function of the excitation wavelength.

**Figure 7:**
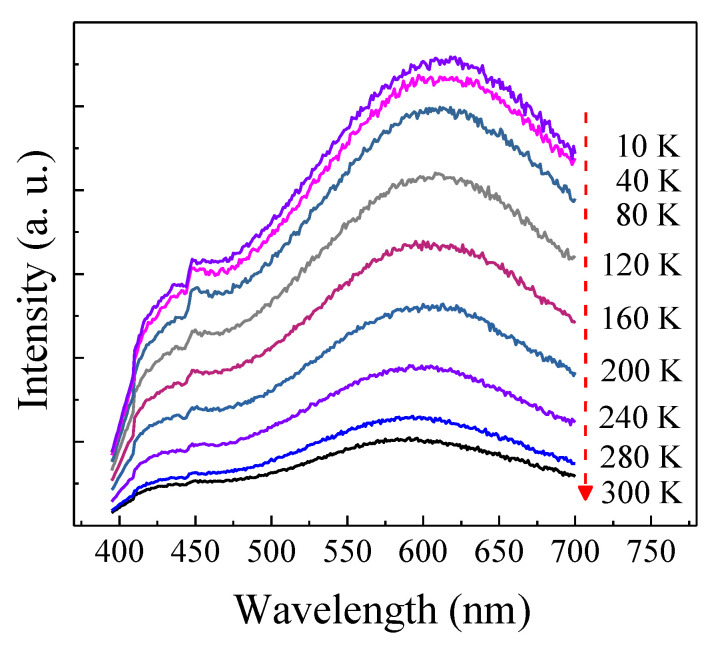
PL spectra measured at different temperatures ranging from 10 K to 300 K.

To further understand the enhanced PL characteristics, PL (570 nm) decay curves were taken from the samples excited by a 372 nm picosecond laser, as illustrated in Figure 5. All the PL decay curves can be fitted by a two-exponent function, as demonstrated in the following [17]:

(1) $$I\left(t\right)=I_{0}+I_{1}\exp\left(\frac{-t}{\tau_{1}}\right)+I_{2}\exp\left(\frac{-t}{\tau_{2}}\right),$$

where *I*_0_ is the background level, and *I*_i_ and *τ*_i_ (*i* = 1, 2) are the corresponding weight fraction and lifetime of each exponential decay component, respectively. The intensity-weighted averaged PL lifetimes are then determined by $\left(A_{1}*\tau_{1}^{2}+A_{2}*\tau_{2}^{2})/(A_{1}*\tau_{1}^{}+A_{2}*\tau_{2}^{}\right)$. As demonstrated in Figure 5, all samples featured a fast decay dynamic with the lifetimes of ~1 ns. Generally speaking, annealing at high temperatures can effectively passivate the surface state of silicon nanostructures to a certain extent, thereby making the PL lifetime longer, as well as producing the PL enhancement. However, the lifetimes in our case were independent of annealing temperatures. This indicated that the enhancement of PL did not come from the improved surface state passivation of Si nanostructures in our case. In fact, such a luminescent dynamic behavior was the same as that observed in the defect-related luminescent a-SiN_x_ [13,14]. According to Robertson and Powell, in the case of a-SiNx, the radiative recombination at Si dangling bonds (K0) would give rise to 570 nm PL, whereas the radiative recombination either from the conduction band to the N_2_^0^ level or the valence band to the N_4_^+^ level would result in ~430 nm PL [18]. Therefore, the improved PL was considered to originate from the increased density of radiative centers related to the Si dangling bonds (K0) and N_4_^+^ or N_2_^0^ as a result of bonding configuration reconstruction, as revealed in Figure 4. To gain more insight into the enhanced PL characteristics, the sample annealed at 1000 °C was excited by different wavelengths, as shown in Figure 6. The PL positions almost showed no recognized changes with increasing excitation wavelength from 280 nm to 340 nm. Furthermore, the PL peak position excited by the 325 nm line was independent of the temperature, as illustrated in Figure 7. These results excluded the possibility that the 570 and 425 nm PL emissions were from band-to-band recombination in Si nanodots, further indicating that the PL arises from defect-related luminescence centers. Therefore, based on the above results, the enhanced emission can be attributed to the increased density of radiative centers related to the Si dangling bonds (K0) and N_4_^+^ or N_2_^0^ in a-SiNx.

## 4. Conclusions

In summary, the effect of thermal annealing on the luminescent a-SiNx containing dense Si nanodots was investigated. Thermal annealing of the films remarkably improved the PL intensity by more than twofold compared with the pristine sample. The PL features a recombination dynamic on a nanosecond timescale. Based on the PL results and the analyses of Raman spectra and the bonding configurations of the samples, the enhanced green emission was attributed to the increased density of radiative centers related to the Si dangling bonds (K0) and N_4_^+^ or N_2_^0^ as a result of bonding configuration reconstruction.

## Data Availability

Data underlying the results presented in this paper are not publicly available at this time but may be obtained from the authors upon reasonable request.
